# Histolytics: A panoptic spatial analysis framework for interpretable histopathology

**DOI:** 10.1016/j.csbj.2025.11.022

**Published:** 2025-11-11

**Authors:** Oskari Lehtonen, Niko Nordlund, Shams Salloum, Ilkka Kalliala, Anni Virtanen, Sampsa Hautaniemi

**Affiliations:** a Research Program in Systems Oncology, Research Programs Unit, Faculty of Medicine, University of Helsinki, Helsinki, Finland; b Department of Pathology, University of Helsinki and HUS Diagnostic Center, Helsinki University Hospital, Helsinki, Finland; c Department of Obsteterics and Gynaecology, University of Helsinki and Helsinki University Hospital, Helsinki, Finland

**Keywords:** Histopathology, Digital pathology, Cancer, Panoptic segmentation, Spatial analysis, Interpretable features, Software, AI, Deep learning, Machine learning

## Abstract

Quantifying spatial organization in hematoxylin and eosin (H&E)–stained whole-slide images (WSIs) is essential for uncovering tissue-level patterns relevant to pathology. We present Histolytics, an open-source, scalable Python framework for interpretable, WSI-scale histopathological analysis. Histolytics integrates panoptic segmentation with spatial querying, morphological profiling, and graph-based analytics to enable high-resolution, quantitative characterization of nuclei, tissue compartments, and the extracellular matrix (ECM). Designed to align with diagnostic reasoning, Histolytics supports segmentation with state-of-the-art deep learning models and provides modular tools for extracting biologically grounded features across entire WSIs. By leveraging spatially contextualized measurements at cellular and tissue levels, Histolytics addresses a critical gap in explainable computational pathology, offering an interpretable alternative or complement to black-box predictive models. We validated Histolytics through segmentation benchmarking on cervical and ovarian high-grade serous carcinoma data.

## Graphical Abstract

**Figure:**
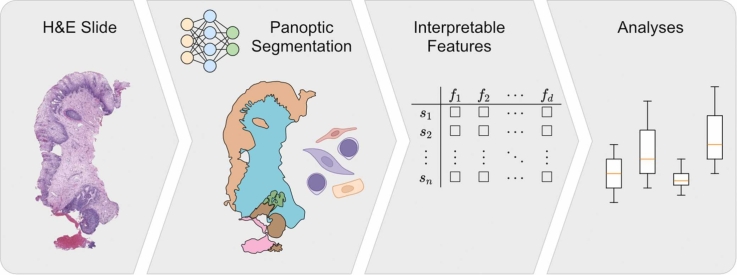

## 1 Introduction

Hematoxylin and Eosin -stained (H&E) tissue whole-slide images (WSIs) form the cornerstone of histopathology. Their rich information content, coupled with their widespread availability, has presented a powerful resource for understanding diseases for centuries. Indeed, histopathology has a long and established history rooted in the visual assessment of features that are directly associated with various disease states, forming the basis of diagnosis and disease behavior. More recently, the advent of deep learning (DL) approaches has introduced a powerful predictive modeling paradigm which has led to significant increases in computational prognostic accuracy, automated grading of tumors, and prediction of treatment response [1], [2], [3], [4], [5]. To increase the acceptance and trust in these artificial intelligence (AI) predictions, improvements in textual and visual explainability have also been rapid [6], [7], [8], [9].

Despite the transformative potential of AI, the adoption of these models in routine histopathology workflows has been slow and applications remain mostly in the research settings [10], [11]. A major limitation of these models is their inability to numerically quantify predefined or novel histology-informed features with biological relevance, such as tissue and cell type compositions, and nuclear and extracellular matrix (ECM) morphology and organization. In contrast, recent methods utilizing segmentation and spatial analysis techniques have shown promise in such prognostics and diagnostics applications [12], [13]. However, while significant efforts have been directed towards frameworks unifying DL-based histological predictive modeling under easy-to-use computational frameworks a similar effort to unify panoptic segmentation based spatial feature quantification methods under a comprehensive open-source framework is still lacking (Supplementary Table S1) [14], [15], [16], [17].

We developed Histolytics, an open-source Python framework designed to extract interpretable features that reflect human understanding and closely align with expert diagnostic reasoning. Histolytics uniquely combines full-slide panoptic segmentation [18], which is a powerful approach to simultaneously segment tissues and nuclei. Unlike semantic or instance-only segmentation, panoptic segmentation provides both cellular and tissue-level delineation in a unified representation, allowing analyses that require understanding of individual nuclei in their precise tissue context. Together with advanced spatial analysis methods enabling interpretable, WSI-scale insights that reflect real-world tissue architecture and diagnostic reasoning. Histolytics also integrates with the wider Python ML ecosystem that together with modular application programming interface (API), detailed documentation, and extensive tutorials, ensure ease of use and integration into diverse computational pathology workflows. Histolytics is available at https://github.com/HautaniemiLab/histolytics with documentation and tutorials at https://hautaniemilab.github.io/histolytics/.

## 2 Results

**Fig. 1:**
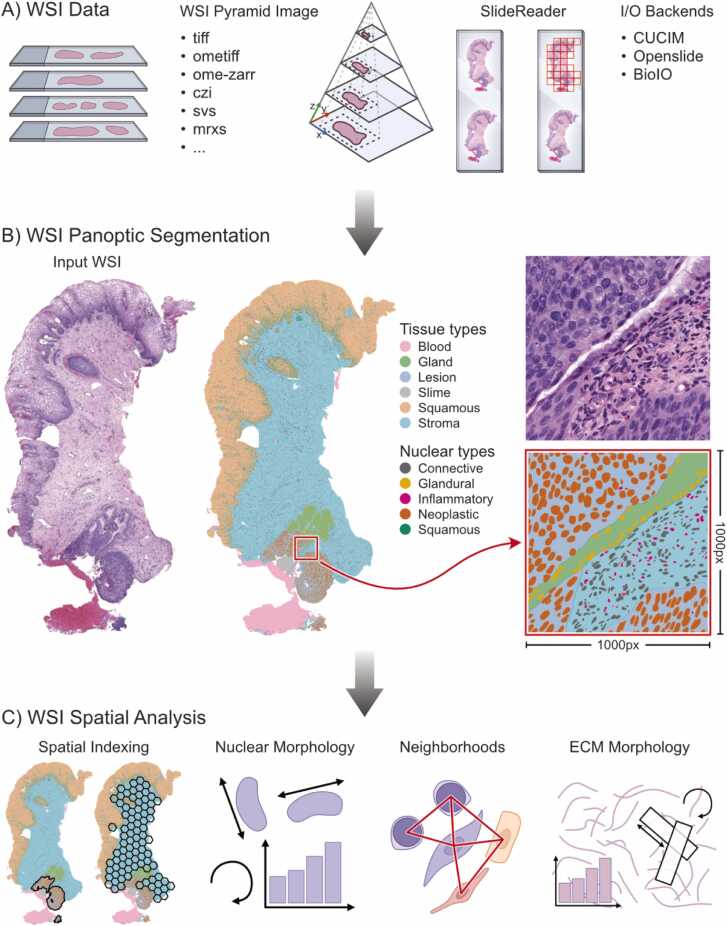
Histolytics is a spatial analysis library for histopathological images based on whole-slide image (WSI)-scale panoptic segmentation. A) Histolytics supports a wide range of WSI data formats through a versatile SlideReader-object with support for mask assisted tiling and a connected components-based tile selector. The SlideReader-object can be initialized with three different backends for WSI Input/Output (I/O): CuCIM, Openslide and BioIO. B) Illustration of WSI-scale panoptic segmentation maps, the primary engine of spatial analysis in Histolytics for spatially aware interpretable histopathological feature extraction. C) Histolytics spatial analysis is built upon a strong spatial indexing system and a wide array of methods for quantification of cell nuclei morphology, neighborhood relationships, and extracellular matrix (ECM) morphology.

The design principle of Histolytics relies on three pillars, versatile WSI Input/Output (I/O), panoptic segmentation, and flexible spatial feature extraction (Fig. 1). WSI I/O, a pre-requisite for panoptic segmentation and downstream spatial analyses at WSI-scale, is handled in Histolytics with a versatile WSI SlideReader-class. The class, through different slide-reading backends, supports an extensive set of slide formats (Supplementary Table S2) [19], [20], [21]. Additionally, it includes slide-level masking and tile-filtering that enable the removal of WSI background and redundant sets of tissues (Fig. 1a) for optimized panoptic segmentation run-times.

To enable panoptic segmentation, Histolytics contains modular implementations of well-established cell segmentation models with tissue segmentation extensions [22], [23], [24], [25]. The modular implementations allow the easy integration of different model backbones such as the most recent histopathological foundation models, allowing users to leverage the latest advancements in deep histological feature representations in their segmentation pipelines [1], [2], [3]. To streamline fine-tuning and deployment, Histolytics offers several pre-trained models (available at https://huggingface.co/histolytics-hub) and a range of training and model validation tools, including multitask losses, regularization techniques, and performance evaluation metrics (Supplementary Table S2). The generalization performance of two panoptic segmentation models used in this study, fine-tuned on cervical and ovarian high-grade serous carcinoma (HGSC) H&E data, was assessed on the PanNuke dataset [26], [27], where the HGSC model achieved the best overall performance (PQ = 0.43, AP = 0.57; Supplementary Table S3).

To enable efficient spatial analysis, Histolytics can merge patch-level segmentations into unified WSI-scale panoptic segmentation maps (Fig. 1b), allowing location-based and morphometric analyses to operate directly at the slide level without need to operate at tile-level. Building on this, Histolytics provides efficient spatial indexing and querying to retrieve objects and tissue regions of interest, enabling spatially localized analyses on WSIs. The framework further includes a comprehensive suite of nuclei-, tissue-feature extraction methods spanning morphological, intensity-based, textural, spatial graph-based, and cluster-level metrics (Fig. 1c; Supplementary Table S2). For analyses that require raw image intensities, Histolytics supports scalable tile-level processing via the WSIPatchIterator-object (Supplementary Fig S1 shows extensive performance and WSI scalability comparisons of Histolytics). Built upon Libpysal [28], Geopandas [29], the familiar pandas DataFrame API [30], and a vectorized segmentation data format (GeoJSON, GeoParquet), Histolytics ensures seamless integration with the broader Python data science and machine learning ecosystem, while allowing the use of widely used image viewers like QuPath for interactive visualization of segmentation and spatial analysis outputs [31].

### 2.1 WSI-scale cellular organization analysis via spatial querying

The quantification of the spatial organization of different cell types within different tissue compartments is fundamental in deciphering biological processes, particularly in complex environments like the tumor microenvironment [32], [33]. For instance, to quantify Tumor Infiltrating Lymphocytes (TILs), a prognostic biomarker in several cancers [34], [35], the cancer and immune cell populations need to be precisely pinpointed within regions of interest. Mapping and counting specific nuclei within regions of interest can be challenging due to the potentially vast number of nuclei present in the WSIs. Histolytics is designed to address this through efficient spatial querying and spatial partitioning tools applied to panoptic segmentation maps.

**Fig. 2:**
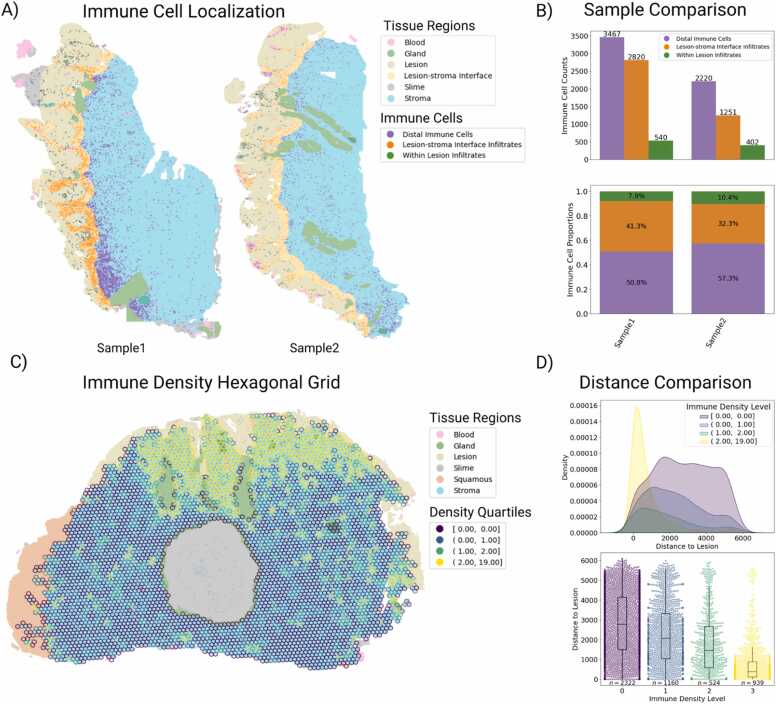
Region-based spatial queries of immune cells and stromal partitioning in segmented cervical biopsy whole-slide images (WSIs). A) Panoptic segmentation of two cervical biopsies followed by downstream stromal partitioning. Stromal regions adjacent to the lesion were defined as the lesion-stroma-interface (LSI), with remaining stroma classified as distal. Immune cells within lesion, LSI, and distal stroma were quantified. This partitioning enabled examination of lymphocyte localization at the lesion boundary, where immune activity often concentrates, allowing the exploration of spatial immune patterns that relate to anti-tumor response. B) Comparison of immune cell distributions across the two samples (absolute counts and proportional differences). In the first biopsy, 49.2 % of all immune cells are located within or adjacent to lesion with especially high concentrations of immune cells located at the LSI (41.3 %). In contrast, in the second biopsy we see less overall infiltration as 42.7 % of the immune cells are located within or adjacent to lesion with 32.3 % located at the LSI, in the second biopsy, indicating less prominent lesion-proximal immune infiltration. C) Application of hexagonal spatial indexing to a segmented cervical biopsy. Immune cell counts are computed per hexagon and binned into four quartiles, enabling localized assessment of immune-rich regions and their spatial distribution relative to the lesion, rather than relying solely on bulk tissue-level counts. D) Distance-to-lesion analysis based on the immune densities of the hexagons. For each hexagonal region, the Euclidean distance from its centroid to the lesion boundary was computed and compared across immune-density bins. Immune-dense hexagons were consistently located closer to the lesion than immune-sparse regions, indicating active immune response in the example.

To demonstrate how Histolytics can be used for quantifying immune cell infiltration at WSI-scale, we used Histolytics’ cervical panoptic segmentation model (hovernet-histo-cin2-pan-v1) and spatial querying tools to compare the levels of immune infiltration in two cervical intraepithelial lesions, a precursor to cervical cancer that is caused by a viral infection which can provoke host immune responses [36]. To add granularity to the analysis, we also used Histolytics’ interface-region tool to partition the segmented stromal tissues into two distinct sectors relative to the lesions: an adjacent zone representing the lesion-stroma interface (LSI) and the distal stromal region (Fig. 2a). This allowed us to compare the immune cell counts and proportions within the lesions, at the interface area, and at the distal stromal regions (Fig. 2b).

To further demonstrate how Histolytics can be used to quantify spatial patterns of immune cell distributions that broad region-based queries might miss, we use Histolytics’ to partition segmented cervical stromal tissue into smaller, uniform units. By using the H3 hierarchical geospatial indexing system (https://h3geo.org/), supported by Histolytics, we subdivided the stromal compartments into hexagonal units and quantified the immune densities within each hexagon (Fig. 2c). By computing the shortest distance from the center of each hexagon to the boundary of the lesion, we could quantitatively assess the spatial distribution of immune dense regions relative to the lesion. In this illustrative example, the immune-dense portions of the stroma localize significantly closer to the lesion (Fig. 2d), suggesting an activated immune response.

To support biological validity and illustrate the panoptic segmentation model’s ability to distinguish lymphocytes from morphologically similar nuclei, we evaluated its performance on a held-out in-house cervical validation dataset, achieving good agreement across neoplastic, inflammatory, and connective nuclei (PQ = 0.71 ± 0.32, 0.69 ± 0.16, and 0.71 ± 0.10, respectively; Supplementary Tables S4-S5, S7-S8). We also provide qualitative overlays of nuclei- and tissue-level predictions alongside expert annotations (Supplementary Fig. S2).

### 2.2 Morphological profiling of nuclei and stroma across WSIs

The study of morphological characteristics of nuclei and the ECM is crucial in histopathology for diagnostics, tumor grading, and predicting disease behavior [37], [38]. For instance, changes in nuclear shape and chromatin patterns are well-established indicators of malignancy but assessed descriptively in routine pathology based on variation in nuclear size, shape, and chromatin distribution [38]. Similarly, the architecture and remodeling of the ECM play a significant role in tumor progression [39]. Quantifying these features numerically at a large scale can provide insights into disease mechanisms and histological heterogeneity, while also offering potential for integration into diagnostic workflows as reproducible complements to pathologist assessment. Histolytics offers over 20 shape quantification metrics and several pixel-intensity-based metrics for nuclear, tissue, and ECM morphology characterization.

**Fig. 3:**
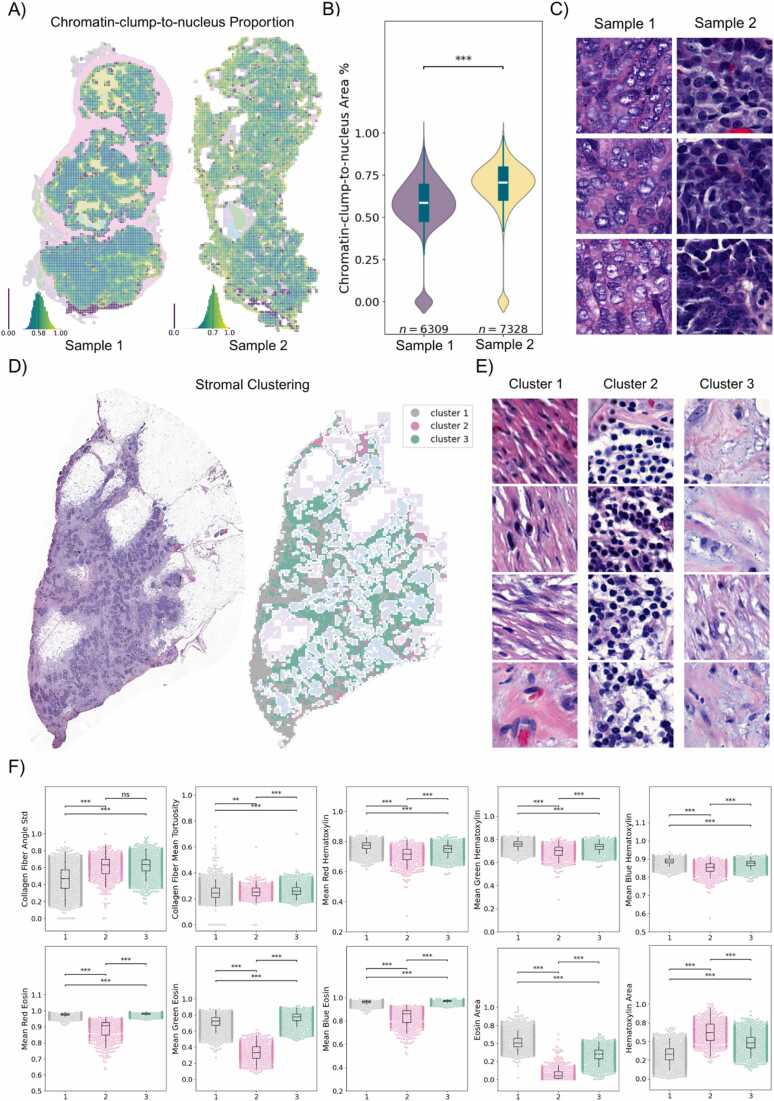
Quantification of morphological features in high grade serous carcinoma (HGSC) omental tumor whole-slide images (WSIs). A) Panoptic segmentation maps of two HGSC omental tumors were generated and tumor regions partitioned into a rectangular grid. To quantify the descriptive chromatin features typical of HGSC, which commonly shows coarse, hyperchromatic nuclei, chromatin clumps were segmented within each nucleus and the mean chromatin-clump-to-nuclear area proportion computed, enabling whole-slide numerical assessment of chromatin distribution within neoplastic nuclei. B) Violin plots comparing the distribution of mean chromatin-clump-to-nuclear area proportions between the two samples, showing statistically significant differences determined by two-sided Mann–Whitney test. The higher proportion in sample 2 corresponds to more uniformly hyperchromatic nuclei, while the other shows a broader range of chromatin distribution, consistent with variable nuclear atypia. C) Close-up examples of nuclei with low and high chromatin-clump-to-nuclear proportions, illustrating the contrast between optically clear and dark hyperchromatic nuclei. D) Ten morphological and intensity-based stromal features were extracted in a HGSC slide and used for unsupervised K-means clustering (k = 3; silhouette-optimized), generating three stromal compartments. This enabled the exploration of differing immune-associated and desmoplastic stromal phenotypes, the latter representing the fibrotic tissue response that develops around invasive tumors. E) Close-up examples of stromal clusters indicative of different maturity levels of reactive stromal desmoplasia and immune density. Cluster 1 represents mature desmoplastic stroma, Cluster 2 represents immune dense stroma, and Cluster 3 represents immature desmoplastic stroma. F) Cluster-wise swarm plots illustrating the min-max normalized stromal features used for clustering, with statistically significant differences between stromal phenotypes (Mann–Whitney tests), illustrating that Histolytics can computationally detect stromal phenotypes typically evaluated descriptively. Mature desmoplastic stroma (Cluster 1) shows increased eosin-associated stain intensity and lower collagen angular deviation; immune-dense stroma (Cluster 2) shows higher hematoxylin stain intensity; and immature desmoplastic stroma (Cluster 3) shows intermediate hematoxylin and eosin stain intensities and a higher collagen angular deviation. The quantified stromal features included collagen orientation variability, collagen tortuosity, stain-intensity measurements for eosin and hematoxylin (six features), and eosin/hematoxylin stain area. P-value annotation legend is the following: *** p < 0.001, ** p < 0.01, * p < 0.05, ns p > 0.05.

To demonstrate Histolytics’ usability for WSI-scale nuclear morphology quantification, we computed the proportion of nuclear area occupied by chromatin for every neoplastic nucleus in two ovarian HGSC slides previously segmented using Histolytics’ HGSC panoptic segmentation model (cellpose-histo-hgsc-pan-v1). By segmenting chromatin clumps within each nucleus, we computed the average proportion of chromatin within nuclei across the slide, allowing us to characterize the WSI-level differences in chromatin patterns between the two slides (Fig. 3a). The first slide in this example shows a broad distribution of optically clear nuclei with peripheral chromatin, whereas the second slide displays uniformly hyperchromatic nuclei across the slide (Fig. 3b, c), reflecting distinct chromatin staining patterns captured by the metric.

To illustrate how Histolytics' can also be used to characterize the morphology and texture of the ECM, we used it to categorize desmoplastic stromal reaction in an omental HGSC slide. Stromal reactions to cancer have variability in their textural characteristics that reflect different stages of stromal maturation and have shown to impact disease progression and therapeutic response [39], [40]. To characterize these maturation levels within the HGSC slide, we used Histolytics to cluster the segmented stroma into three distinct compartments based on ten quantitative morphological and textural features (Fig. 3d). This clustering-based analysis allowed us to dissect the stroma with histopathologic interpretability: Mature fibrotic desmoplasia, immune cell enriched stroma, and immature desmoplastic stroma were represented by clusters 1, 2, and 3, respectively (Fig. 3e), with clear cluster-level feature differences (Fig. 3d).

In sum, Histolytics' comprehensive suite of nuclear and ECM morphology characterization tools showcase its ability to interpretably quantify morphological and textural heterogeneity at the WSI-level. To ensure the accuracy of these illustrative examples, we also evaluated nuclar segmentation performance on held-out HGSC dataset and included expert-annotated overlays demonstrating close agreement in identifying tumor, connective, and inflammatory nuclei (PQ = 0.53 ± 0.11, 0.51 ± 0.16, and 0.59 ± 0.20, respectively; Supplementary Tables S6, S9; Supplementary Fig S3).

### 2.3 Cell and ECM organization through graph-based neighborhood analysis

**Fig. 4:**
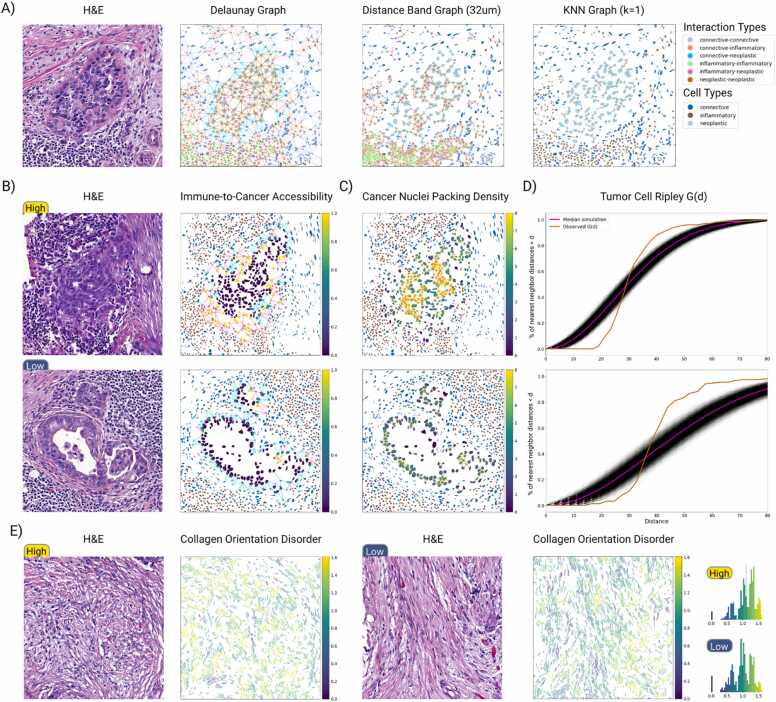
Extraction and quantification of graph-based neighborhood features in high grade serous carcinoma (HGSC) omental tumor images A) Examples of graph algorithms included in Histolytics, namely, Delaunay, Distband (32 μm), and KNN (K=1). B) Immune-to-Cancer accessibility score, a metric derived by computing the proportion of immune cells to connective cells for each neoplastic nuclei neighborhood (Delaunay graph). In the first HGSC example patch, neoplastic nuclei at the tumor-stroma-interface have visibly more immune-to-neoplastic links compared to the second example, where connective cells effectively block the access of immune cells to neoplastic cells, indicating immune-evasive characteristics. C) Neighborhood-based neoplastic cell density (Distband 32 μm), where the first example shows a densely packed solid tumor nest and the second shows a less-dense papillary-like pattern, illustrating how Histolytics can quantify growth-pattern differences typically assessed visually. D) Ripley G-function for the neoplastic cell centroids: the cumulative distribution of nearest neoplastic neighbor distances measured over increasing distance thresholds compared to a set of simulated spatially random poisson processes. The solid tumor nest shows a rapid early rise characteristic of clustering, whereas the papillary region shows a delayed rise consistent with more dispersed architecture. E) Collagen fiber neighborhood orientation disorder (Distband 64 μm), binned via Fisher–Jenks classification. Highly disordered collagen in the first patch reflects irregular and variably oriented fibers, whereas the second patch shows aligned fibers with lower disorder. By enabling whole-slide-scale, automated computation of collagen disorder, Histolytics provides a reproducible quantitative stromal metricthat has been associated with more aggressive disease and poorer outcomes in some tumors [42], [43].

The analysis of spatial relationships between different cellular and ECM components is critical for understanding complex biological processes. In cancer, for example, the interplay between tumor cells and immune cells, as well as the influence of stromal cells like cancer-associated fibroblasts (CAFs) have been shown to impact therapeutic response through immune evasion [41]. Similarly, the remodeled spatial arrangement of collagens in the ECM can influence cell migration, affecting progression in many cancers [42], [43]. Spatial graphs provide a powerful framework for defining proximity-based relationships and establishing spatial neighborhoods for segmented objects. Histolytics supports several spatial graph algorithms (Supplementary Table S2) to define spatial neighborhoods, such as Delaunay triangulation, Distband graphs, and K-nearest neighbor graphs (Fig. 4a). Histolytics also includes commonly used Ripley statistics [44], and global/local spatial autocorrelation (Moran's I [45]) tools to study spatial point patterns and clustering. It also supports custom link-based metrics, enabling flexible analysis of proximity-based neighborhoods.

To showcase Histolytics’ utility for graph-based neighborhood analysis, we used Histolytics to compute a potential surrogate marker for immune evasion for HGSC tumor nests by measuring the level of stromal cells physically blocking the access of immune cells to tumor. For this, we developed a simple single-cell-level metric: immune-to-cancer accessibility score, for which we computed the proportion of immune cells relative to stromal cells within each neoplastic neighborhood. To define the neoplastic neighborhoods, we used a Delaunay graph (maximum link distance of 50μm). Illustrative examples of tumor nests with relatively high and low accessibility scores are shown to visually demonstrate how stromal architecture can either permit or restrict immune proximity to cancer cells (Fig. 4b).

As a further demonstration of Histolytics’ graph-based neighborhood analyses, we quantified the nuclear packing density in two HGSC tumor nests at tile-level. To quantify this spatial metric related to tumor growth patterns, we defined the neoplastic cell neighborhoods using a Distband graph (32μm radius) and computed the number of neoplastic nuclei in each neighborhood. As expected, the solid tumor nest showed higher packing density than the adeno-papillary nest (Fig. 4c). We also measured the level of the neoplastic nuclei clustering using Ripley’s G-function over a range of distance thresholds (Fig. 4d). The more rapid increase of the G-function for the solid growth pattern, indicative of a dense clustered spatial distribution, verified the same observation in the example.

Histolytics can also be used for the graph-based neighborhood analysis of the ECM. As an example, we use Histolytics to investigate the organization of the extracellular matrix by quantifying collagen fiber orientation disorder, a prognostic factor within the tumor microenvironment known to influence cell migration and invasion [42], [46]. For this measurement, we first used Histolytics to segment the collagen fibers from the ECM and defined collagen neighborhoods with a Distband graph (64μm radius). After this, we computed the neighborhood angular diversities using Shannon entropy [47], representing the orientation disorder metric. In the illustrative example, the high collagen disorder sample exemplifies a more chaotic collagen neighborhood, associated with more aggressive disease and poorer outcomes [42], [43], while the low disorder sample exemplifies more aligned collagen fibers (Fig. 4e).

Collectively, these examples demonstrate that Histolytics delivers a flexible platform for in-depth neighborhood analysis, encompassing a variety of graph methods to define neighborhoods, and extensive neighborhood analysis tools for diverse use-cases ranging from prognostic feature extraction to exploratory discovery of spatial features.

### 2.4 Analysis of immune cell clusters across WSIs

The spatial organization of immune cells within the tumor microenvironment, particularly the formation of lymphoid aggregates (Lagg) and tertiary lymphoid structures (TLS), is increasingly recognized as a critical factor influencing anti-tumor immunity and patient prognosis across various cancers, by serving as sites for immune cell activation and differentiation, impacting the local and systemic immune response to the tumor [48], [49]. Further, TLSs have been linked to improved response to immune-checkpoint therapy in many cancers [50], [51]. To detect these clusters, Histolytics supports multiple spatial clustering algorithms and cluster measures for cluster feature analysis (Supplementary Table S2)

**Fig. 5:**
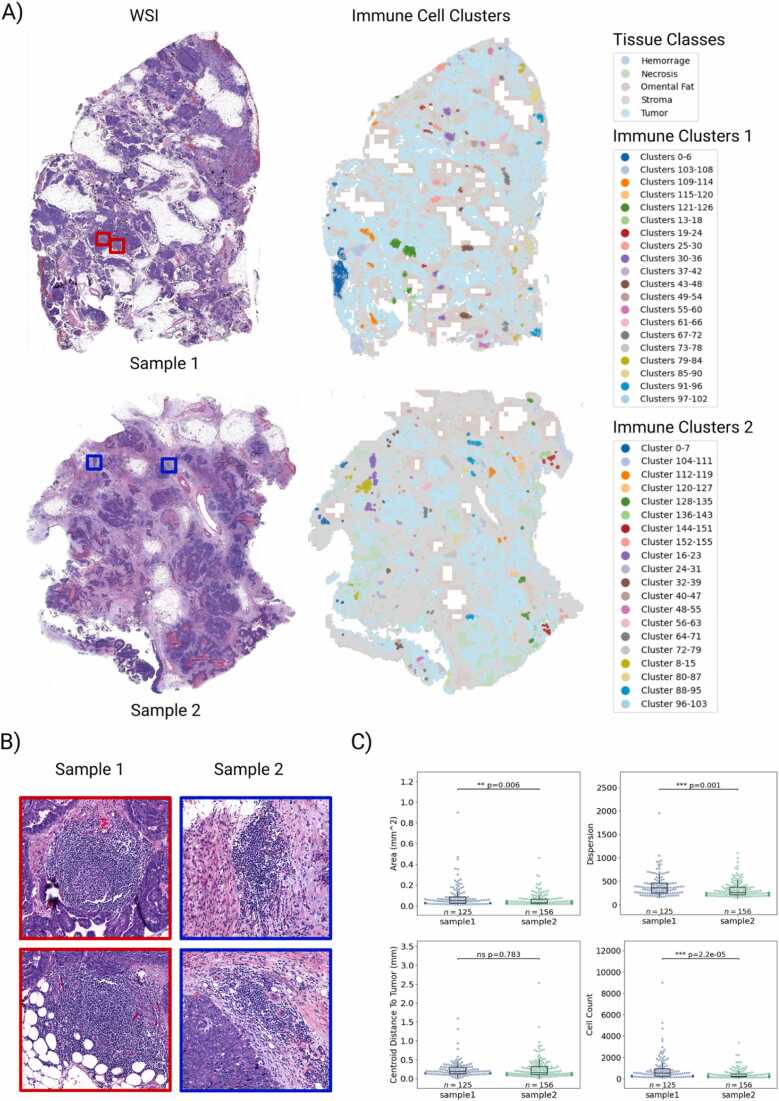
Spatial clustering of immune cells and quantification of cluster-level features in high grade serous carcinoma (HGSC) omental tumor whole-slide images (WSIs). A) Two HGSC omental tumor slides and their corresponding tissue segmentation maps with spatially clustered immune cell aggregations highlighted, representing lymphoid aggregates (Laggs) and tertiary lymphoid structures (TLSs) that reflect immune responses within the tumor microenvironment. The spatial immune cell clusters were found using the DBSCAN algorithm (maximum distance=125 mm, minimum size=100). B) Sample-wise close-up examples of the clusters, detected through spatial clustering. TLSs have been associated with improved anti-tumor immune activity and favorable immunotherapy responses [50], [51] . C) Sample-level comparison of immune cell cluster features. We computed the area, cell count, dispersion (spread), and centroid distance to tumor for each immune cluster in both samples. The first example exhibits significantly larger and more dispersed immune clusters with higher area, dispersion, and cell counts than the second example (Mann–Whitney test), indicating a more active immune response and potential formation of TLS-like aggregates. .

To investigate the spatial clustering of immune cells in HGSC, we leveraged Histolytics to perform WSI-scale cluster analysis on two H&E-stained HGSC samples. We employed density-based spatial clustering algorithm DBSCAN [52], supported by Histolytics, to identify cellular clusters across entire WSIs (Fig. 5a). Immune clusters representing different-sized immune cell concentrates such as Laggs and TLS (Fig. 5b) were detected from both samples. To quantify more specific cluster characteristics, we computed the total area, the number of cells, dispersion and the distance from each cluster to the nearest segmented tumor region for each cluster (Fig. 5c). The first example exhibited, on average, larger and more dispersed immune cell clusters, possibly indicating a larger number of activated lymphoid structures and more pronounced immune activity.

In conclusion, this example demonstrates how Histolytics can be used to quantify immune-cell aggregation patterns to detect large lymphocyte clusters such as Laggs and TLS that have been associated with potential immunotherapy response, highlighting Histolytics’ potential as a scalable framework for screening and stratifying patients based on immune organization relevant to immunotherapy outcomes.

## 3 Discussion

Histolytics is an efficient and scalable framework for comprehensive, interpretable analysis of H&E-stained whole-slide images (WSIs), enabling processing hundreds of slides efficiently. By tightly integrating panoptic segmentation with spatial analysis tools, such as morphological quantification, spatial querying, graph-based neighborhood analysis, and clustering, Histolytics enables consistent extraction of biologically meaningful, spatially contextualized features across diverse tissue types.

To illustrate the utility of Histolytic, we have provided several case studies that illustrate how Histolytics can be applied across multiple levels of tissue organization, from single-cell chromatin patterns to whole-slide immune architecture. These demonstrations highlight three central strengths of the framework. First, the ability to quantify subtle nuclear morphological features, such as chromatin distribution, shows how Histolytics can translate visually assessed microscopic cues into reproducible numerical measurements across entire slides. Second, stromal and extracellular-matrix analyses demonstrate how complex tissue compartments that are traditionally evaluated qualitatively can be systematically decomposed into quantitative descriptors, enabling more consistent examination of tumor-stroma interactions. Third, the graph-based spatial analyses and immune-cluster detection examples illustrate how cellular neighborhoods and immune architecture can be quantified at scale from routine H&E slides, opening opportunities for spatial metrics that may hold prognostic or immunotherapy-response prediction value. Together, these case studies provide a roadmap for how interpretable computational features derived from histological images can support mechanistic investigation, biomarker discovery, immuno-oncology profiling, and reproducible measurements of the ECM with potential diagnostic and prognostic relevance.

We also have shown how Histolytics can facilitate rapid development and testing of new spatial metrics, by defining the immune-to-cancer accessibility score, highlighting how flexible, graph-based neighborhood methods in Histolytics can accelerate hypothesis generation and exploratory biomarker discovery. In conclusion, by standardizing segmentation outputs and spatial feature computation, Histolytics provides a foundation for assisting discovery and validation of biomarkers. Histolytics is freely available with thorough documentation.

## 4 Conclusions

Histolytics introduces an efficient and scalable framework for comprehensive, interpretable analysis of H&E-stained whole-slide images (WSIs), enabling processing hundreds of slides efficiently. By tightly integrating panoptic segmentation with spatial analysis tools, such as morphological quantification, spatial querying, graph-based neighborhood analysis, and clustering, Histolytics enables consistent extraction of biologically meaningful, spatially contextualized features across diverse tissue types. The framework is designed to translate H&E images to knowledge, providing insights that are both quantitatively robust and human interpretable. With its modular design, state-of-the-art segmentation models, and seamless integration into the Python data science ecosystem, Histolytics lowers the barrier to large-scale spatial tissue analysis. Extensive documentation, tutorials, and pretrained models further support wide adoption, making Histolytics a practical tool for advancing transparent, scalable computational pathology.

## 5 Materials & methods

### 5.1 WSI I/O

#### 5.1.1 SlideReader

Whole-slide images in Histolytics are accessed using the SlideReader object, an extension of the HistoPrep library that we adapted to provide a unified interface across multiple vendor formats (Supplementary Table S2). SlideReader integrates three backend engines, cuCIM [19], OpenSlide [20], and BioIO [21], allowing both efficient GPU-accelerated access where supported and broad compatibility with proprietary formats. This architecture ensures stable data loading across heterogeneous datasets.

SlideReader supports extraction of image regions at arbitrary pyramid levels, enabling access to WSI data at different effective magnifications for both high-resolution nuclear analysis and lower-resolution tissue context. In addition to tiled reading, SlideReader includes optional preprocessing utilities for automated tissue detection using Otsu-based thresholding (Scikit-image implementation [53]) and connected-component grouping to isolate contiguous tissue areas in multi-specimen slides [54]. These functions standardize input prior to segmentation and spatial analysis. Code examples are provided in the Supplementary Methods.

### 5.2 WSIPatchIterator

For analyses that require access to raw pixel intensities, Histolytics includes the WSIPatchIterator, a scalable tile-iteration engine designed for efficient whole-slide traversal. Because whole-slide images cannot be loaded into memory in their entirety, this component streams image regions on demand and processes them tile by tile, making it well suited for tasks such as color-based measurements, intensity-driven feature extraction, and image-domain quality control. The iterator supports multi-processing to accelerate patch extraction across large slides and high-resolution magnifications, enabling practical WSI-scale computation in settings where pixel-level access is required rather than polygon-based processing. Code example is provided in the Supplementary Methods.

### 5.3 Panoptic segmentation

#### 5.3.1 Model architectures

Histolytics includes a suite of deep learning architectures designed for panoptic segmentation of histopathology whole-slide images. These models are built on established nuclei segmentation backbones including Cellpose [22], StarDist [23], Hover-Net [24], and Cell-ViT [25]. To support panoptic output, each architecture is extended with an additional semantic segmentation branch that learns to delineate broader tissue regions at the same time as segmenting individual nuclei. This joint prediction strategy enables simultaneous instance-level and tissue-level segmentation from a single model.

We provide several pre-trained models, easily accessible through our Hugging Face model pages (https://huggingface.co/histolytics-hub). All models use pre-trained backbone weights from the PyTorch Image Models (timm) library [55], and are fine-tuned on histopathology datasets to adapt to H&E images. We provide a tutorial (https://hautaniemilab.github.io/histolytics/user_guide/seg/backbones/) demonstrating how various panoptic segmentation models can be initialized with different backbones, including foundation model backbones.

#### 5.3.2 WsiPanopticSegmenter

Whole-slide panoptic segmentation in Histolytics is performed using the WsiPanopticSegmenter-object, which orchestrates tiled inference and reconstruction of full-resolution segmentation maps. The workflow operates on regions extracted by the SlideReader and applies a pre-trained panoptic segmentation model tile-by-tile across the whole-slide image.

After segmentation, tile-level outputs are merged into a unified whole-slide segmentation map, with optional geometry simplification and precision control to balance file size and contour fidelity. This approach enables efficient processing of large histopathology slides while preserving fine-grained nuclear and tissue boundaries.

A tutorial (https://hautaniemilab.github.io/histolytics/user_guide/seg/panoptic_segmentation/) on how to run WSI-level panoptic segmentation and merging is provided in Histolytics documentation pages, showcasing how users can efficiently process large histopathological images to generate comprehensive, whole-slide-level panoptic segmentation maps.

#### 5.3.3 Model Training and Benchmarking Utilities

To support training and evaluation of panoptic segmentation models, Histolytics includes utility functions that manage multi-task optimization and model assessment. The training module implements commonly used segmentation loss functions (Supplementary Table S2) and a multi-task-losses that enable joint optimization of instance- and tissue-level predictions with configurable weighting. Regularization strategies, including StrongAugment [56], are provided to improve generalization in limited-data settings. Users can fine-tune existing models on new datasets or train models from the beginning, with detailed workflows available in Histolytics online documentation at (https://hautaniemilab.github.io/histolytics/user_guide/seg/finetuning/).

For performance evaluation, Histolytics includes a comprehensive set of standard metrics used in nuclei and tissue segmentation research. These include panoptic quality (PQ) and average precision (AP) for instance segmentation, and intersection over union and DICE coefficients for pixel-level tissue classification (Full set of metrics in Supplementary Table S2).

### 5.4 Spatial analysis

#### 5.4.1 Spatial querying

Histolytics includes an efficient spatial querying module that enables extraction of segmented cellular and tissue objects based on their spatial relationships. The querying engine uses R-tree spatial indexing through the Geopandas library to rapidly subset nuclei, stromal cells, or other segmented structures within user-defined regions of interest or within specific tissue compartments [29]. This allows targeted analysis of biologically relevant areas, such as tumor boundaries, stromal zones, or immune-rich regions. Examples are provided in the Supplementary Methods and an online tutorial (https://hautaniemilab.github.io/histolytics/user_guide/spatial/querying/).

#### 5.4.2 Spatial partitioning

Histolytics offers flexible methods for spatially partitioning tissue segmentations. The library implements buffering-based techniques to delineate interface regions between distinct tissue types. By applying a user-defined buffer around the boundary of one tissue region, overlapping areas with an adjacent tissue can be extracted and analyzed, enabling the study of interactions and transitions at tissue interfaces.

Furthermore, Histolytics provides functionality to partition tissue regions into regular spatial grids, including hexagonal grids and rectangular grids. With these grids, tissue regions can be subdivided into smaller, uniform units, allowing for the quantification of spatial features such as cell densities, morphological characteristics, or marker expression within each grid cell, enabling the extraction of localized patterns at WSI-scale.

Tutorial (https://hautaniemilab.github.io/histolytics/user_guide/spatial/partitioning/) on spatial partitioning are also available, where we show how segmented tissue regions can be partitioned for more localized analysis

#### 5.4.3 Chromatin clump segmentation

To characterize intranuclear chromatin organization, Histolytics includes a chromatin segmentation module that identifies dense chromatin aggregates within each nucleus. The method applies multi-Otsu thresholding (Scikit-image implementation [53]) to the grayscale image, restricted to the nuclear mask, to separate compact chromatin from more diffusely stained nuclear regions [54]. The resulting binary chromatin maps enable quantification of chromatin distribution patterns at scale. Example usage is provided in the Supplementary Methods.

#### 5.4.4 Nuclear features

Histolytics provides a comprehensive suite of quantitative nuclear features to characterize nuclear morphology and chromatin architecture at scale. The feature library includes measures of nuclear geometry and size, grayscale and color-intensity characteristics, textural descriptors, and metrics describing the distribution of chromatin clumps (Full list in Supplementary Table S2). Together, these features capture key aspects of nuclear pleomorphism, ranging from shape irregularity and nuclear enlargement to chromatin condensation and textural heterogeneity.

Nuclear features are computed directly from segmented nuclei and their corresponding image regions, producing per-nucleus measurements suitable for downstream statistical and spatial analyses. Detailed usage examples and full feature definitions are available in the Supplementary Methods and online documentation (https://hautaniemilab.github.io/histolytics/user_guide/spatial/nuclear_features/).

#### 5.4.5 Collagen fiber segmentation

To support quantitative analysis of extracellular matrix architecture, Histolytics includes a module for automated collagen fiber segmentation from H&E images. The approach applies Canny edge detection (Scikit-image implementation [53]) to the grayscale image to highlight fibrillar structures, followed by restriction of edge detection to stromal regions to exclude background and highly eosinophilic non-fibrous tissue [57]. This produces a binary representation of collagen fibers suitable for downstream extraction of fiber-level and stromal organizational features. A Code example is provided in the Supplementary Methods.

#### 5.4.6 Stromal features

Histolytics provides quantitative descriptors of extracellular matrix morphology, including features derived from segmented collagen fibers and stromal intensity profiles. Fiber-based features characterize structural attributes such as length, orientation, curvature, and tortuosity of collagen strands (Supplementary Table S2), enabling assessment of stromal organization and remodeling.

In addition, Histolytics extracts stromal intensity features using HED color-decomposed H&E (Scikit-image implementation [53]) images to isolate hematoxylin- and eosin-rich stromal components. After separating stain channels through established color-deconvolution methods, Otsu thresholding is used to identify eosinophilic and hematoxylinophilic stromal areas, excluding cellular and background regions [54]. Intensity distributions and stain-occupancy measures are then computed to characterize stromal composition. We provide a tutorial (https://hautaniemilab.github.io/histolytics/user_guide/spatial/stromal_features/) showcasing how users can efficiently characterize stromal features in their H&E images.

#### 5.4.7 Graphs

Histolytics leverages graph-based representations to quantify neighborhood relationships and spatial interactions between segmented objects. In a graph, a segmented object is a node, and a link between two nodes is defined by a graph algorithm and a distance threshold (links can be formed between nodes that are spatially close to each other). A node and all its links to other nodes form a local neighborhood.

In Histolytics, the graph implementations rely on the Libpysal library and supported graph types include, but are not limited to, Delaunay triangulation, distance-based graphs (Distband), and KNN-graphs (full list in Supplementary Table S2) [28]. Users can specify distance thresholds to restrict connections to biologically meaningful spatial scales, allowing flexible modeling of tumor–immune interactions, local tissue microenvironments, and cellular packing patterns. This graph representation enables subsequent computation of neighborhood-level features and spatial statistics. Examples of graph creation and graph centrality feature extraction workflows are provided in the online documentation (https://hautaniemilab.github.io/histolytics/user_guide/spatial/graphs/).

#### 5.4.8 Neighborhood features

Histolytics includes tools to quantify and aggregate characteristics around each nucleus or a segmented object (local spatial neighborhoods). For each object, summary statistics of neighboring entities can be computed within graph-defined or distance-based neighborhoods, allowing aggregation of feature values across immediate spatial contexts. The framework also supports calculation of pairwise distances among neighboring objects, enabling quantitative assessment of local spacing and proximity relationships. In addition, several standard diversity indices, including Simpson [58], Shannon [47], Gini [59], and Theil indices [60], can be computed to characterize heterogeneity within local neighborhoods. These operations provide a flexible basis for downstream spatial analyses, such as cell packing density or local compositional variation of nuclear types and angular diversity of chromatin fibers. We provide a comprehensive tutorial in Histolytics online documentation (https://hautaniemilab.github.io/histolytics/user_guide/spatial/nhoods/), showcasing the usage of neighborhood feature extraction.

#### 5.4.9 Ripley statistics

Ripley functions are a group of spatial statistics that focus on how to characterize clustering in point patterns through pairwise and nearest neighbor distances [44]. Ripley functions characterize point patterns as a function of distance with the aim of determining whether the point patterns are random, dispersed, or clustered. Histolytics contains implementations of Ripley’s K, L and G functions.

Ripley’s K-function estimates the expected number of points within a certain distance of a randomly chosen point. By calculating this across a range of distances, the K-function returns a vector of cumulative average number of points lying within a given distance of a typical data point. The Ripley’s L-function is a transformation of the K-function that stabilizes the variance of the K-estimator and transforms it into a straight line making the visual assessment of the function easier.

The Ripley’s *G-*function, on the other hand, summarizes the distribution of the nearest neighbor distances between each point in a point pattern. This is done by computing the proportion of points for which the nearest neighbor is within a given distance for a range of distance thresholds. Ripley’s G-function returns a vector of cumulative percentages against increasing distances.

In Histolytics, Ripley statistics can be computed directly from segmented point coordinates and evaluated against spatially random reference distributions to determine whether observed spatial patterns differ from randomness across distance scales. This implementation enables users to assess clustering or dispersion characteristics in point patterns in a statistically rigorous manner. Example usage is provided in the Supplementary Methods.

#### 5.4.10 Global and local spatial autocorrelation

Spatial autocorrelation refers to the degree to which features or values are correlated to each other across space. Positive spatial autocorrelation indicates that nearby objects tend to have similar values, while negative spatial autocorrelation suggests that nearby objects tend to have dissimilar values. To quantify spatial autocorrelation, Histolytics implements Moran's I statistic which can be used to assess whether the distribution of a specific feature's values across the segmented objects is spatially random, clustered, or dispersed.

Histolytics supports both global and local Moran’s I statistics through implementations provided by the Libpysal library [28]. These tools evaluate whether spatial variation in a given feature differs from random expectation, either across the entire slide or within specific local neighborhoods. Global Moran’s I provides an overall measure of spatial autocorrelation, while local Moran’s I highlights regions that contribute to spatial clustering or dispersion. Example usage is provided in the Supplementary Methods.

#### 5.4.11 Spatial Clustering and Centrography

Histolytics supports spatial clustering of segmented objects using two complementary strategies. Density-based clustering methods (Supplementary Table S2), including DBSCAN and related approaches (Scikit-learn implementation [61]), are used to detect spatially compact cell clusters directly from coordinates [52].

The obtained clusters can be characterized quantitatively using measures of cluster size, spatial extent, orientation, and dispersion, as well as distances to relevant tissue regions. These descriptors facilitate systematic analysis of spatial clustering patterns at WSI-scale. We provide a tutorial showcasing the usage of density-based clustering and cluster feature extraction methods in Histolytics documentation pages (https://hautaniemilab.github.io/histolytics/user_guide/spatial/clustering/)

#### Code Availability

Histolytics is distributed as a pip-installable Python package, and the source code is accessible in the GitHub repository: https://github.com/hautaniemilab/histolytics. Comprehensive user guides and API reference are provided in the accompanying documentation: https://hautaniemilab.github.io/histolytics/. The analytical workflows necessary to reproduce the findings presented in this manuscript are integrated within the WSI Analysis Workflows section of the User Guide documentation pages https://hautaniemilab.github.io/histolytics/user_guide/.

## CRediT authorship contribution statement

**Oskari Lehtonen:** Conceptualization, Methodology, Software, Validation, Formal analysis, Investigation, Data Curation, Visualization, Writing – Original Draft, Writing – Review & Editing. **Niko Nordlund:** Software, Methodology, Data Curation, Writing – Review & Editing. **Shams Salloum:** Data Curation, Writing – Review & Editing. **Ilkka Kalliala:** Resources, Writing – Review & Editing, Funding acquisition. **Anni Virtanen:** Resources, Conceptualization, Supervision, Project administration, Validation, Writing – Review & Editing, Funding acquisition. **Sampsa Hautaniemi:** Resources, Conceptualization, Supervision, Project administration, Writing – Review & Editing, Funding acquisition.

## Declaration of Competing Interest

The authors of the manuscript **Histolytics: A panoptic spatial analysis framework for interpretable histopathology** have declared no conflict of interest.

## Data Availability

To facilitate the reproducibility of the analyses presented in this manuscript, processed datasets are made available through the histolytics.data module. The provided segmentation and image data are sufficient to fully replicate and demonstrate the analytical workflows described herein. The original image data from which these segmentations were derived originated from the Helsinki University Hospital (HUS), and the DECIDER cohort (Multi-layer Data to Improve Diagnosis, Predict Therapy Resistance and Suggest Targeted Therapies in HGSOC; ClinicalTrials.gov identifier: NCT04846933). Details regarding the preprocessing and panoptic segmentation of the original image data can be found within the segmentation pages of the Histolytics user guide documentation https://hautaniemilab.github.io/histolytics/user_guide/.

## Footnotes

Appendix A Supplementary data associated with this article can be found in the online version at doi:10.1016/j.csbj.2025.11.022.
