# Supplementary material for: Histolytics: A panoptic spatial analysis framework for interpretable histopathology
Source: Comput Struct Biotechnol J. 2025 Nov 11;27:5135–47. doi: 10.1016/j.csbj.2025.11.022 (PMC12664990; doi:10.1016/j.csbj.2025.11.022)
Supplement: Supplementary file 1 — Supplementary material [file mmc1.docx]

## Histolytics - Supplementary

Oskari Lehtonen^1^, Niko Nordlund^1^, Shams Salloum^1^, Ilkka Kalliala^3^, Anni Virtanen^1,2,*^, Sampsa Hautaniemi^1,*^

^1^Research Program in Systems Oncology, Research Programs Unit, Faculty of Medicine, University of Helsinki, Helsinki, Finland;

^2^Department of Pathology, University of Helsinki and HUS Diagnostic Center, Helsinki University Hospital, Helsinki, Finland;

^3^Department of Obsteterics and Gynaecology, University of Helsinki and Helsinki University Hospital, Helsinki, Finland

* To whom correspondence should be sent: Professor Sampsa Hautaniemi ([sampsa.hautaniemi@helsinki.fi](mailto:sampsa.hautaniemi@helsinki.fi)) and Dr Anni Virtanen ([anni.virtanen@hus.fi](mailto:anni.virtanen@hus.fi)).

# Supplementary Figures


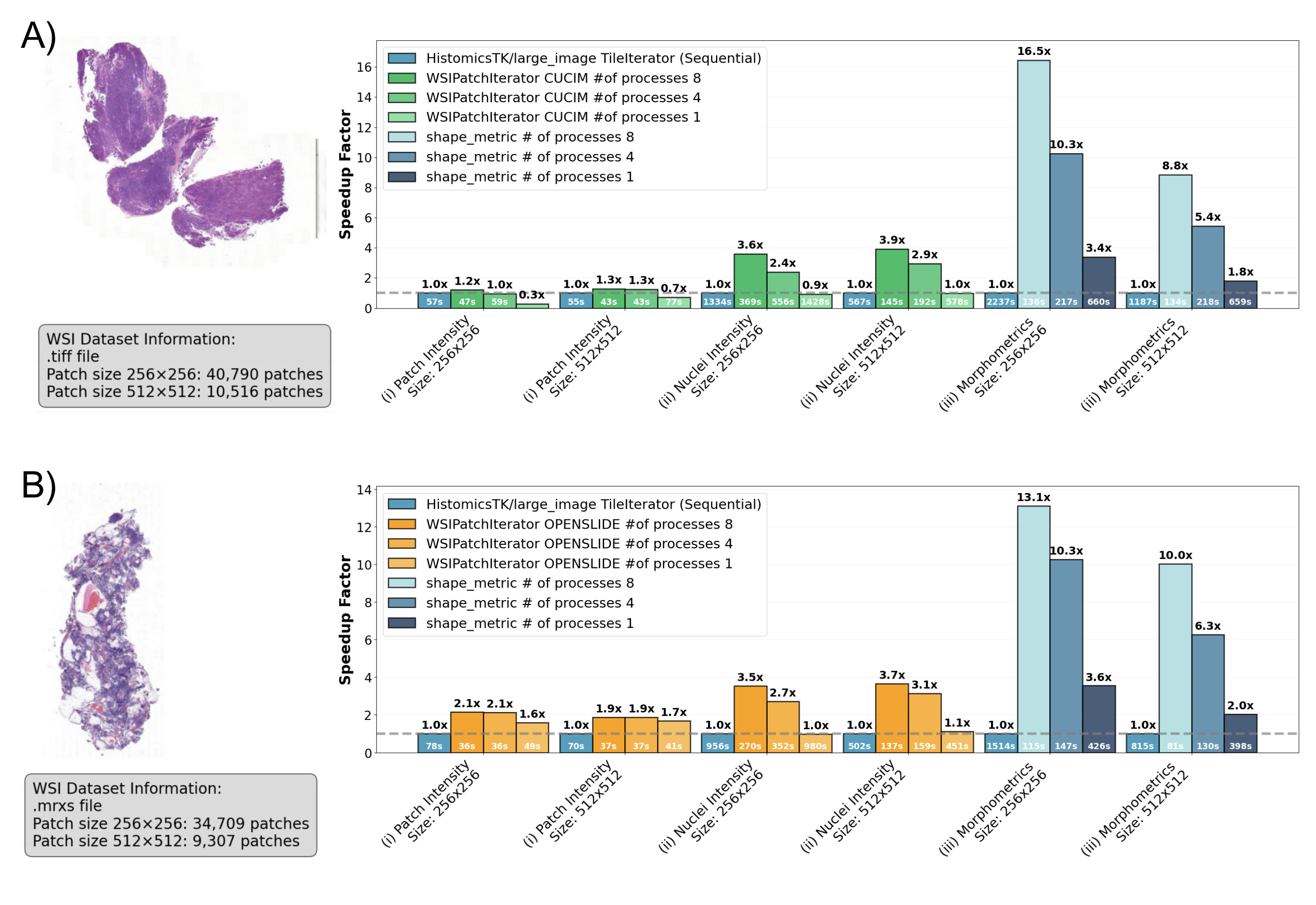
**Supplementary Figure S1:** **Benchmarking and scalability of Histolytics whole-slide-image (WSI) patch iteration and feature extraction compared to HistomicsTK/large_image package.** Grouped barplots display the relative performance gain of Histolytics over the HistomicsTK/large_image package for three representative analysis tasks [1]. Absolute runtimes (in seconds) are annotated within each bar for reference. The benchmarked tasks were: (i) patch-level intensity mean computation, (ii) nuclei grayscale intensity computation, and (iii) nuclei morphometric feature extraction (computed directly from the nuclei segmentation map in Histolytics using shape_metric function, whereas HistomicsTK requires tile iteration with the TileIterator object). Two patch sizes (256×256px and 512×512px) were evaluated for TIFF and MIRAX whole-slide image formats (.tiff and .mrxs) to assess scalability across resolutions and WSI file formats. For the .tiff file, 40,790 patches (256×256) and 10,516 patches (512×512) were processed; for the .mrxs file, 34,709 patches (256×256) and 9,307 patches (512×512) were processed. The patches were extracted at 20x magnification. A) .tiff benchmark. Histolytics with CuCIM backend shows clear speed ups across all tasks. For patch-level intensity computation, speed-ups reach approximately 1.3× for both patch sizes when using 8 processes. For nuclei grayscale intensity computation, speed-ups increase to 3.6× (256 × 256) and 3.9× (512 × 512). For morphometric extraction, bypassing additional tile traversal yields the largest gains, reaching 16.5× and 8.8× against HistomicsTK tile iteration-based computation with 256 × 256 and 512 × 512 tiles respectively. B) .mrxs benchmark. Improvements follow a similar pattern. Histolytics with Openslide backend achieves approximately 2.3× (256 × 256) and 1.9× (512 × 512) speed-ups with 8 processes for patch-level intensity computation. Nuclei grayscale intensity computation reaches 3.5× (256 × 256) and 3.7× (512 × 512). Morphometric extraction again yields largest performance advantages bypassing additional tile traversal, reaching 13.1× and 10.0× against HistomicsTK tile iteration-based computation with 256 × 256 and 512 × 512 tiles respectively.

Benchmarks were run using: 11th Gen Intel(R) Core(TM) i9-11950H @ 2.60GHz CPU with 8 physical cores (16 logical cores)


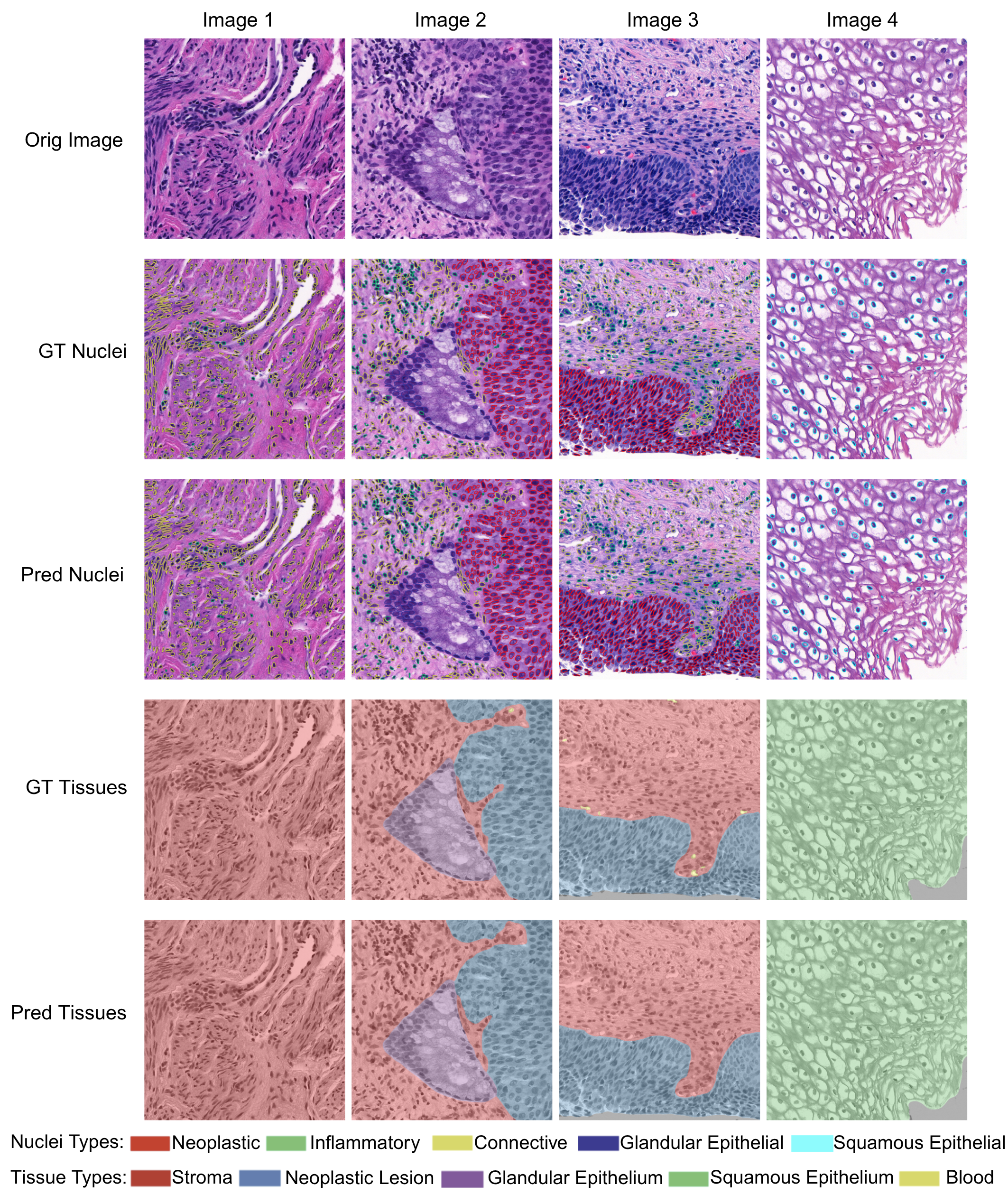


**Supplementary Figure S2: Visual comparison of predicted and ground-truth (GT) nuclei and tissue segmentations on representative patches from the in-house cervical validation dataset**. The predictions were generated using the cervical panoptic segmentation model hovernet-histo-cin2-pan-v1 from the Histolytics model hub. Three cervical H&E tiles (1024x1024px, 20x magnification) are shown (columns), each displayed with the original H&E image, nuclei segmentation overlays, and tissue-level segmentation overlays (rows). The first example (Image 1) contains tangentially sectioned fibroblasts within the stromal compartment, demonstrating the model’s ability to distinguish inflammatory nuclei from stromal fibroblasts despite their similar round hyperchromatic appearance. The second and third examples include neoplastic lesion regions with adjacent stromal components, showing accurate delineation of neoplastic epithelium, stromal tissue, and detection of immune infiltrates. The fourth example includes mature squamous epithelium, also demonstrating good segmentation of this tissue type.


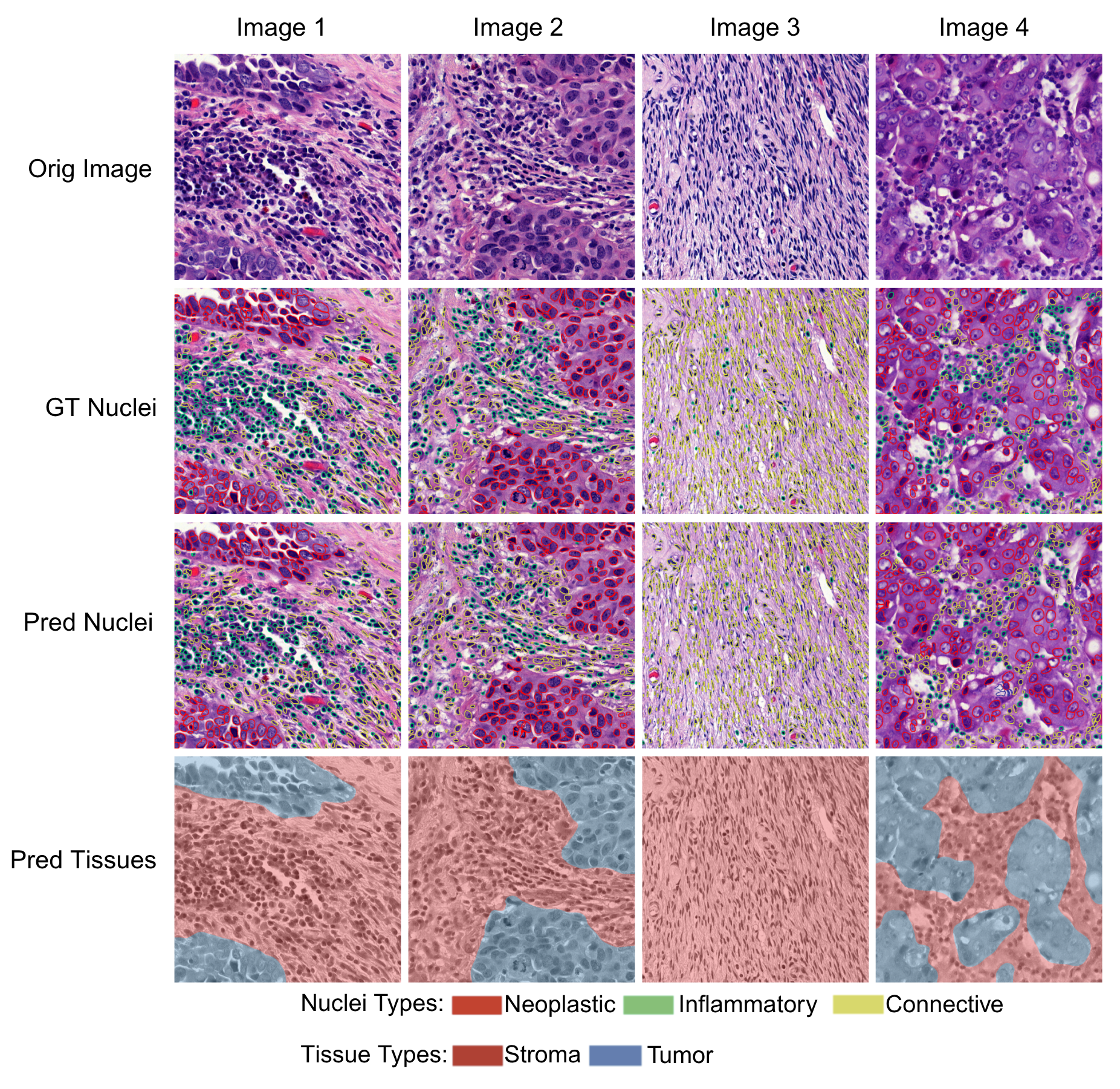
**Supplementary Figure S3: Visual comparison of predicted and ground-truth (GT) nuclei segmentations on representative patches from the in-house private high grade serous carcinoma (HGSC) validation dataset**. The predictions were generated using the cervical panoptic segmentation model cellpose-histo-hgsc-pan-v1 from the Histolytics model hub. Four HGSC H&E tiles (1024x1024px, 20x magnification) are shown (columns), each displayed with the original image and nuclei segmentation overlays (rows). We also provide the predicted tissue-level segmentation overlays, however, the validation set does not contain ground truth tissue type annotations for visual comparison. All the examples demonstrate good delineation of connective, tumor, and inflammatory nuclei in HGSC tissue.


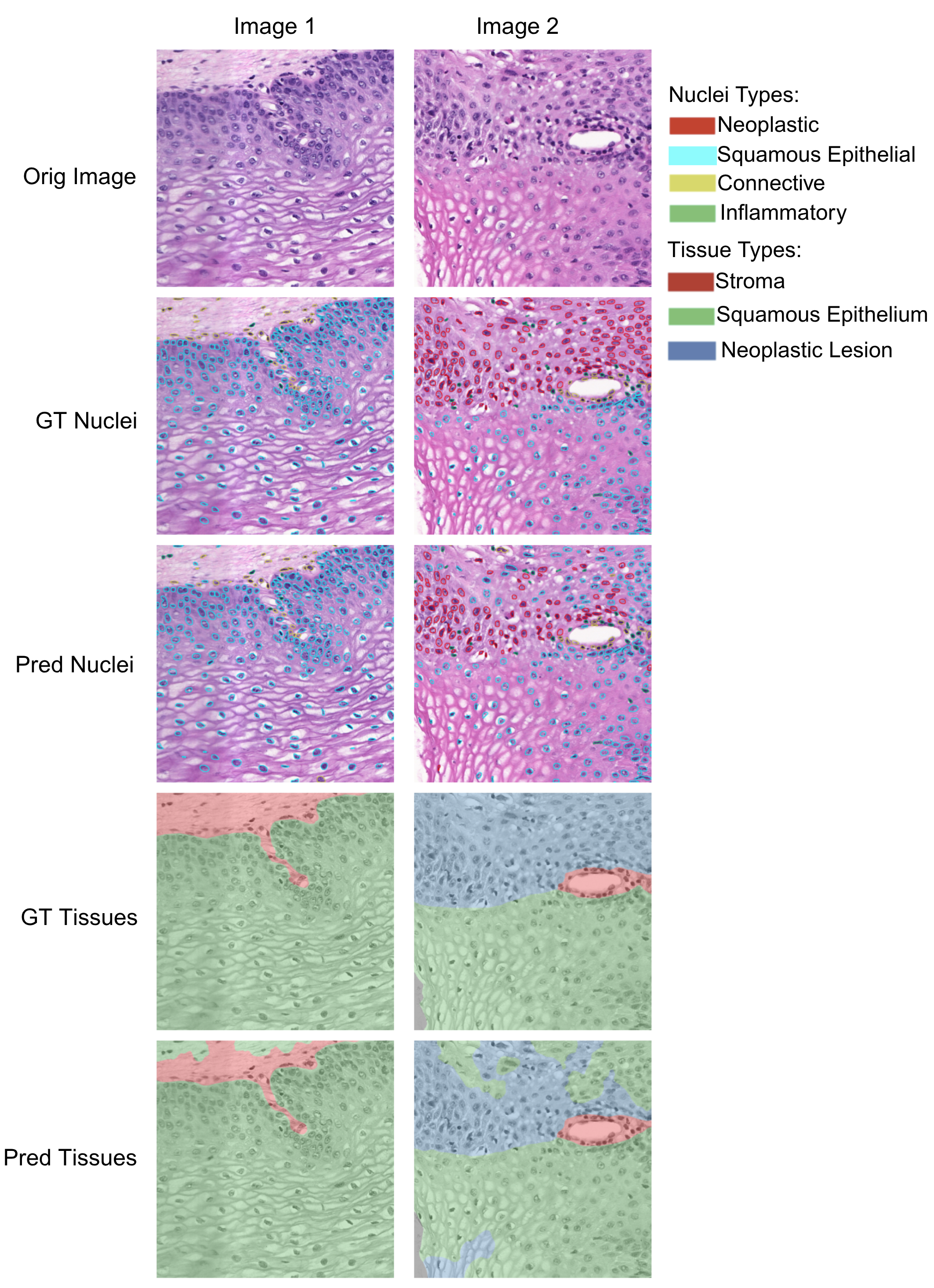


**Supplementary Figure S4: Examples illustrating segmentation inaccuracies of the cervical panoptic segmentation model.** Representative comparisons of predicted and ground-truth (GT) nuclei and tissue segmentations from the in-house cervical validation dataset. Predictions were generated using the Histolytics model hovernet-histo-cin2-pan-v1. Two cervical H&E tiles (1024×1024 px, 20× magnification) are shown, each displayed with the original H&E image, model predictions, and corresponding expert annotations. Both examples demonstrate segmentation inaccuracies observed in challenging regions, including partial misclassification between neoplastic lesion and stroma, and squamous epithelial and stromal nuclei in the first example. In the second example misclassification between healthy squamous epithelium and neoplastic nuclei and tissues are seen.


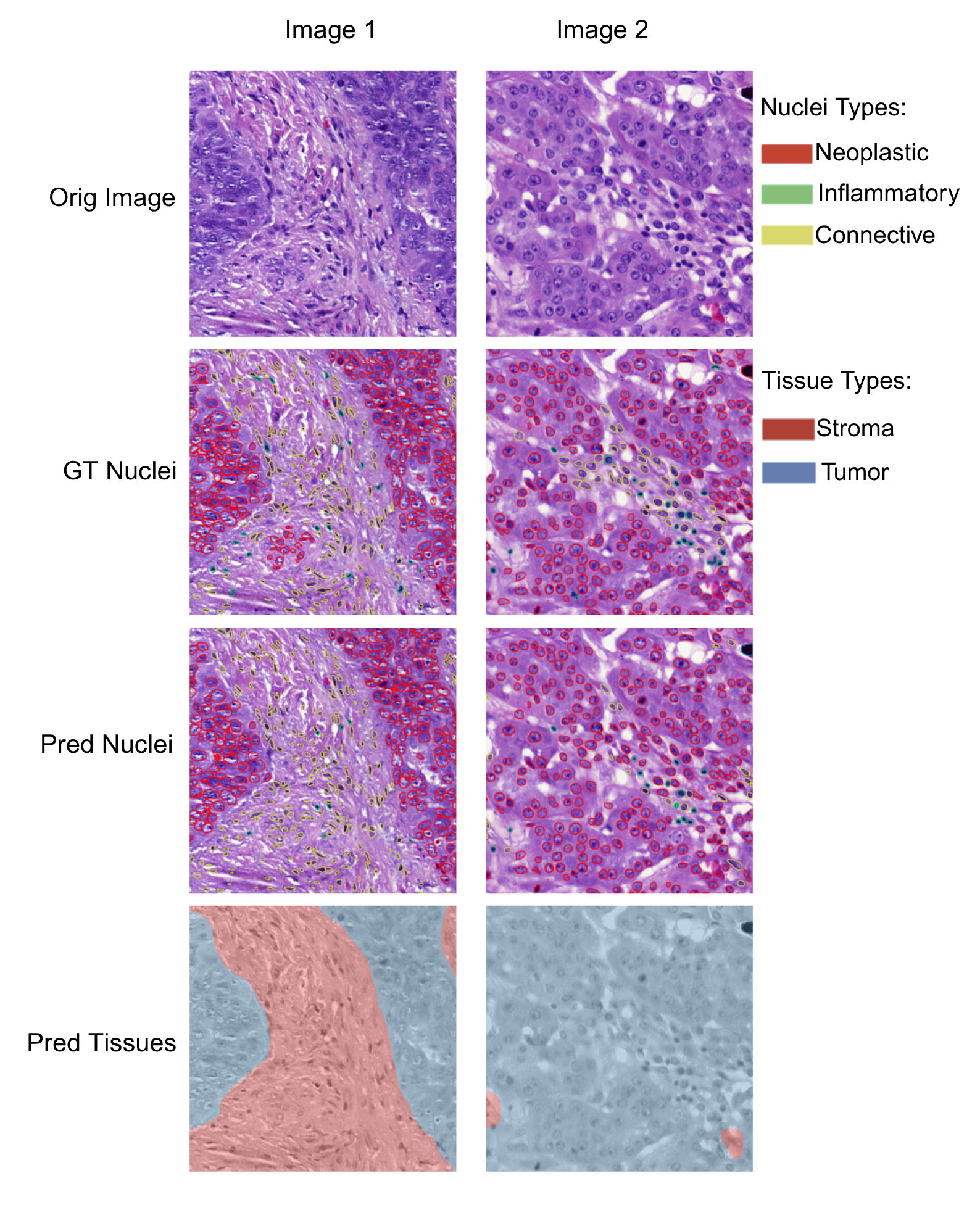


**Supplementary Figure S5: Examples illustrating segmentation inaccuracies of the high grade serous carcinoma (HGSC) panoptic segmentation model.** Representative comparisons of predicted and ground-truth (GT) nuclei and tissue segmentations from the in-house HGSC validation dataset. Predictions were generated using the Histolytics model cellpose-histo-hgsc-pan-v1. Two HGSC H&E tiles (1024×1024 px, 20× magnification) are shown, each displayed with the original image and nuclei segmentation overlays (rows). We also provide the predicted tissue-level segmentation overlays, however, the validation set does not contain ground truth tissue type annotations for visual comparison. Both examples demonstrate segmentation inaccuracies observed in challenging regions, including partial misclassification between neoplastic epithelium and stroma and neoplastic nuclei and stromal nuclei.

# Supplementary Tables

**Supplementary Table S1:** **Qualitative comparison of feature coverage across Histolytics and other computational pathology frameworks:** The table summarizes the availability of key functionalities in nine open-source toolkits for digital pathology and spatial image analysis: Histolytics, TIAtoolbox [2], SlideFlow [3], PathML [4], SquidPY [5], SCIMAP [6], HIPS [7], PHARAOH [8], and HistomicsTK [1]. Columns represent the individual software frameworks, and rows list the supported features grouped into thematic categories: Histopathology focus and WSI I/O, Panoptic segmentation capabilities (including training, benchmarking, pre-trained models, and model merging), Spatial analysis tools (querying and partitioning), Nuclear-level features (morphometrics, intensity, texture, chromatin, etc.), Stromal features (collagen and ECM metrics), Graph- and neighborhood-based features, Spatial clustering, Visualization, Documentation, and Software engineering attributes (testing, packaging, and open-source status). Each entry indicates whether the functionality is natively supported (Yes), partially supported through external tools, or absent.

**Supplementary Table S2: Functional components and feature categories implemented in Histolytics.**
 The table lists the different features, algorithms, and analytical functions available within the Histolytics framework, covering segmentation, feature extraction, spatial analysis, clustering, and WSI I/O capabilities.

| **Nuclei Segmentation Benchmark - Pannuke** | | | | | | | |
| --- | --- | --- | --- | --- | --- | --- | --- |
| **Algorithm** | **Pixel-level** | | | **Instance Level** | | | |
|  | **Accuracy** | **F1-score** | **IoU** | **PQ** | **DQ** | **SQ** | **AP** |
| HistomicsTK max_clustering | 0.87 ± 0.12 | 0.59 ± 0.17 | 0.43 ± 0.16 | - | - | - | - |
| HistomicsTK detect_nuclei_kofahi | 0.87 ± 0.13 | 0.59 ± 0.18 | 0.44 ± 0.18 | - | - | - | - |
| HistomicsTK gaussian_voting | 0.84 ± 0.11 | 0.47 ± 0.13 | 0.31 ± 0.11 | - | - | - | - |
| HistomicsTK gvf_tracking | 0.87 ± 0.12 | 0.60 ± 0.16 | 0.45 ± 0.15 | - | - | - | - |
| QuPath WatershedCellDetection | 0.82 ± 0.13 | 0.55 ± 0.17 | 0.39 ± 0.15 | - | - | - | - |
| Histolytics Hover-Net (histo-cin2-pan-v1) | 0.90 ± 0.09 | 0.48 ± 0.28 | 0.36 ± 0.24 | 0.29 ± 0.19 | 0.58 ± 0.24 | 0.42 ± 0.27 | **0.59 ± 0.30** |
| Histolytics Cellpose (histo-hgsc-pan-v1) | **0.92 ± 0.06** | **0.71 ± 0.18** | **0.57 ± 0.18** | **0.43 ± 0.17** | **0.70 ± 0.14** | **0.59 ± 0.22** | 0.57 ± 0.23 |

**Supplementary Table S3:** **PanNuke nuclei segmentation: pixel- and instance-level performance**. Nuclei segmentation performance on the PanNuke benchmark [9,10], reporting both pixel-level segmentation quality (Accuracy, F1-score, IoU) and overall instance-level segmentation quality (PQ, DQ, SQ, AP). Classical nuclei segmentation methods from HistomicsTK and QuPath are compared with two Histolytics models [1,11]. Histolytics implementations of Cellpose (histo-hgsc-pan-v1) and Hover-Net (histo-cin2-pan-v1) demonstrate moderate overall performance on this multi-tissue benchmark, with the Cellpose HGSC model achieving the highest overall instance- and pixel-level scores. While the HGSC model shows reasonable generalization across tissue types, the cervical model performs less robustly outside its native training domain and is better suited for analyses of cervical tissue. Dashes indicate metrics that were not available for the corresponding baseline methods.

| **Nuclei Segmentation Benchmark - Cervix** | | | | |
| --- | --- | --- | --- | --- |
|  | **PQ** | **SQ** | **DQ** | **AP** |
| **Neoplastic** | 0.71 ± 0.32 | 0.76 ± 0.34 | 0.79 ± 0.35 | 0.79 ± 0.35 |
| **Inflammatory** | 0.69 ± 0.16 | 0.84 ± 0.15 | 0.8 ± 0.18 | 0.86 ± 0.16 |
| **Connective** | 0.71 ± 0.1 | 0.87 ± 0.03 | 0.82 ± 0.1 | 0.83 ± 0.12 |
| **Glandular Epithelial** | 0.76 ± 0.2 | 0.85 ± 0.2 | 0.85 ± 0.22 | 0.88 ± 0.21 |
| **Squamous Epithelial** | 0.67 ± 0.3 | 0.77 ± 0.32 | 0.75 ± 0.34 | 0.76 ± 0.34 |

**Supplementary Table S4:** **Nuclei instance segmentation performance metrics on in-house cervix validation dataset.** The predictions were generated using the cervical panoptic segmentation model hovernet-histo-cin2-pan-v1 from the Histolytics model hub. Metrics include Panoptic Quality (PQ), Segmentation Quality (SQ), Detection Quality (DQ), and Average Precision (AP). Each value is presented as the mean ± standard deviation across cell types.

| **Tissue Segmentation Benchmark - Cervix** | | |
| --- | --- | --- |
|  | **Dice** | **IoU** |
| **Blood** | 0.26 ± 0.38 | 0.22 ± 0.34 |
| **Neoplastic Tissue** | 0.92 ± 0.23 | 0.9 ± 0.23 |
| **Glandular Epithelium** | 0.96 ± 0.02 | 0.93 ± 0.04 |
| **Slime** | 0.86 ± 0.35 | 0.85 ± 0.35 |
| **Squamous Epithelium** | 0.97 ± 0.04 | 0.94 ± 0.07 |
| **Stroma** | 0.84 ± 0.28 | 0.79 ± 0.28 |

**Supplementary Table S5:** **Performance metrics for cervix tissue semantic segmentation on in-house cervix validation dataset** The predictions were generated using the cervical panoptic segmentation model hovernet-histo-cin2-pan-v1 from the Histolytics model hub. Metrics include Intersection over Union (IoU) and Dice Coefficient (Dice). Each value is presented as the mean ± standard deviation across segmentation types.

| **Nuclei Segmentation Benchmark - HGSC** | | | | |
| --- | --- | --- | --- | --- |
|  | **PQ** | **SQ** | **DQ** | **AP** |
| **Connective** | 0.53 ± 0.11 | 0.82 ± 0.03 | 0.65 ± 0.13 | 0.621 ± 0.14 |
| **Inflammatory** | 0.51 ± 0.16 | 0.83 ± 0.04 | 0.61 ± 0.18 | 0.74 ± 0.18 |
| **Neoplastic** | 0.59 ± 0.2 | 0.78 ± 0.23 | 0.70 ± 0.23 | 0.69 ± 0.24 |

**Supplementary Table S6:** **Performance metrics for HGSC nuclei instance segmentation on in-house HGSC validation dataset.** The predictions were generated using the ovarian panoptic segmentation model cellpose-histo-hgsc-pan-v1 from the Histolytics model hub. Metrics include Panoptic Quality (PQ), Segmentation Quality (SQ), Detection Quality (DQ), and Average Precision AP). Each value is presented as the mean ± standard deviation across cell types

| **Nuclei – Cervix Validation Data Statistics** | |
| --- | --- |
| **Cell Type** | **Annotated Nuclei Count (%)** |
| Neoplastic | 6,986 (31.3%) |
| Inflammatory | 3,619 (16.2%) |
| Connective | 5,878 (26.3%) |
| Glandular Epithelial | 3,100 (13.9%) |
| Squamous Epithelial | 2,770 (12.4%) |
| Total | 22,353 |

**Supplementary Table S7: Nuclei counts for manually annotated validation set of cervix data.** Summary of annotated nuclei counts across different cell types in Cervix validation dataset containing 41 1024x1024px H&E image tiles at 20x magnification. Nuclei types: connective (stromal), inflammatory cells, neoplastic cells, glandular epithelium, and squamous epithelium.

| **Tissue – Cervix Validation Data Statistics** | |
| --- | --- |
| **Tissue Type** | **Pixel Count for Tissue Type** |
| Stroma | 16,507,014 (24.6%) |
| Neoplastic lesion | 14,578,060 (21.8%) |
| Squamous Epithelium | 15,220,187 (22.7%) |
| Glandular structures | 10,351,796 (15.5%) |
| Slime | 8,513,648 (12.7%) |
| Blood | 878,817 (1.3%) |
| Total | 66,997,100 |

**Supplementary Table S8: pixel counts of tissues for manually annotated validation set of cervix data.** Summary of manually annotated pixel counts for different tissue types in Cervix validation dataset containing 41 1024x1024px H&E image tiles at 20x magnification. Tissue types: Stroma, neoplastic lesion, squamous epithelium, glandular epithelium, slime, blood

| **Nuclei – HGSC Validation Data Statistics** | |
| --- | --- |
| **Cell Type** | **Annotated Nuclei Count** |
| Neoplastic | 10,679 (49.7%) |
| Inflammatory | 3,978 (18.5%) |
| Connective | 6,837 (31.8%) |
| Total | 21,494 |

**Supplementary Table S9: Nuclei counts for manually annotated validation set of HGSC data.** Summary of annotated nuclei counts across different nuclei types in HGSC validation dataset containing 52 1024x1024px H&E image tiles at 20x magnification. Cell types: neoplastic, connective (stromal), inflammatory nuclei. The validation dataset does not contain tissue type annotations.

## **Code Examples**

This section provides consolidated code examples illustrating the use of core Histolytics modules for WSI I/O, panoptic segmentation, feature extraction, spatial querying, and spatial neighborhood analysis. These examples accompany the methodological descriptions in the main text and serve as practical usage references for reproducing the workflows demonstrated in this study.

### **WSI I/O and Tissue Masking**

from histolytics.wsi.slide_reader import SlideReader
from histolytics.wsi.utils import get_sub_grids

reader = SlideReader(path="slide.svs", backend="CUCIM")

# Tissue mask
thresh, tissue_mask = reader.get_tissue_mask(level=0)

# Tile coordinates for tissue regions
coords = reader.get_tile_coordinates(width=512, tissue_mask=tissue_mask)
connected_tile_grids = get_sub_grids(coords)
filtered_tiles = [grid for grid in connected_tile_grids if len(grid) > 100]

### **WSI-Level Panoptic Segmentation**

from histolytics.wsi.wsi_segmenter import WsiPanopticSegmenter

from histolytics.wsi.slide_reader import SlideReader
from histolytics.models.cellpose_panoptic import CellposePanoptic

reader = SlideReader(path="slide.svs", backend="CUCIM")

# Load model from model hub
model = CellposePanoptic.from_pretrained("hgsc_v1_efficientnet_b5")

model = model.set_inference_mode()
segmenter = WsiPanopticSegmenter(reader, model, level=0, coordinates=coords)

# Run WSI-level panoptic segmentation
segmenter.segment("out/tiles/")

# Merge nuclei and tissue segmentations
segmenter.merge_instances("out/tiles/nuc", dst=”out/nuc_merged.parquet”)

segmenter.merge_tissues("out/tiles/tissue", dst=”out/tis_merged.parquet”)

### **WSI Tile Iteration**

import numpy as np

from histolytics.wsi.slide_reader import SlideReader

from histolytics.wsi.wsi_iterator import WSIPatchIterator

reader = SlideReader(path="slide.svs", backend="CUCIM")

thresh, tissue_mask = reader.get_tissue_mask(level=-1) # extract tissue

coords = reader.get_tile_coordinates(width=256, tissue_mask=tissue_mask)

patch_loader = WSIPatchIterator(reader, coords, batch_size=8, num_workers=8)

with patch_loader as loader:

for batch_idx, batch in enumerate(loader):

means = np.mean(batch, axis=(1, 2)) # compute patch mean

### **Nuclei-Level Feature Extraction**

Import geopandas as gpd

from histolytics.spatial_geom.shape_metrics import shape_metric
from histolytics.nuc_feats.intensity import grayscale_intensity

from histolytics.nuc_feats.chromatin import chromatin_feats

from histolytics.nuc_feats.texture import textural_feats
from histolytics.data import hgsc_cancer_he, hgsc_cancer_inst_mask # example data

# Compute morphometrics for nuclei
morphometrics = shape_metric(nuclei_gdf, metrics=["area","eccentricity"])

img = hgsc_cancer_he()

Label_mask = hgsc_cancer_inst_mask()

# Compute grayscale intensity features

gray_feats = grayscale_intensity_feats(img, label_mask)

# Compute chromatin distribution features
chrom_feats = chromatin_feats(img, label_mask, metrics=["chrom_area"])

# Compute textural features
texture_feats = textural_feats(img, label_mask, metrics=["contrast"])

### **Chromatin Clump Segmentation**

from histolytics.nuc_feats.chromatin import extract_chromatin_clumps

from histolytics.data import hgsc_cancer_he, hgsc_cancer_inst_mask # example data

img = hgsc_cancer_he()

label_mask = hgsc_cancer_inst_mask()

chrom_mask = extract_chromatin_clumps(img, label_mask)

**Graph Construction and Neighborhood Features**

from histolytics.spatial_graph.graph import fit_graph
from histolytics.neighborhood import local_character, local_distances, local_diversity

from histolytics.data import cervix_nuclei # Example data

nuc = cervix_nuclei()

# Fit Delaunay graph to nuclei
w, w_gdf = fit_graph(nuc, method="delaunay", threshold=50)

# Compute mean neighbor distances for each nuclei

nuc = local_distances(nuc, w, reductions=["mean"])

# Compute the mean and std of mean neighbors distance for each nuclei neighborhood
nuc = local_character(nuc, w, val_cols=["nhood_dists_mean”], reductions=["mean", "std"])

# Compute the simpson diversity index for the mean neighbor distances for each nuclei neighborhood

nuc = local_diversity(nuc, w, val_cols=["nhood_dists_mean”], metrics=["simpson_index"])

### **Ripley Statistics**

from histolytics.spatial_clust.ripley import ripley_test
from histolytics.data import cervix_nuclei # Example data

nuc = cervix_nuclei()

# Compute ripley G for distances 50 and 100px
ripl, sim, pvals = ripley_test(nuc, distances=[50, 100], ripley_alphabet="g")

### **Local and Global Spatial Autocorrelation**

from histolytics.data import hgsc_cancer_nuclei

from histolytics.spatial_clust.autocorr import local_autocorr

from histolytics.spatial_graph.graph import fit_graph

from histolytics.utils.gdf import set_uid

from histolytics.spatial_geom.shape_metrics import shape_metric

# Load the HGSC cancer nuclei dataset

nuc = hgsc_cancer_nuclei()

neo = nuc[nuc["class_name"] == "neoplastic"]

neo = set_uid(neo)

neo = shape_metric(neo, ["area"])

# Fit a spatial graph to the neoplastic nuclei

w, _ = fit_graph(neo, "distband", threshold=100)

# Calculate local Moran's I for the area feature

pval, moran_i, quadrants = local_autocorr(neo, w, feat="area")

### **Spatial Clustering and Cluster Features**

from histolytics.spatial_clust.density_clustering import density_clustering

from histolytics.spatial_clust.cluster_metrics import cluster_feats

from histolytics.data import cervix_nuclei # Example data

nuc = cervix_nuclei()

# Cluster immune nuclei

immune_nuc = nuc[nuc[“class_name”] == “inflammatory”]

labels = density_clustering(immune_nuc, eps=350, min_samples=50)

immune_nuc[“label] = labels

# Compute cluster features (area, dispersion, size, orientation)

cluster_features = cluster_feats(immune_nuc)

### **Collagen Fiber Segmentation and ECM Features**

from histolytics.seg import extract_collagen_fibers
from histolytics.features import fiber_feats, stromal_intensity_feats

# Extract collagen mask
collagen_mask = extract_collagen_fibers(img, label_mask)

# Compute collagen features
collagen_feats = fiber_feats(img, metrics=["average_turning_angle", "tortuosity"])

# Compute stromal intensity features
stromal_feats = stromal_intensity_feats(img, label_mask)

### **Spatial Querying**

from histolytics.spatial_ops.ops import get_objs

from histolytics.data import cervix_nuclei, cervix_tissue # Example data

tissue = cervix_tissue()

nuc = cervix_nuclei()

# Extract nuclei within stroma

stroma_roi = tissue[tissue[“class_name”] == “stroma”]
nuclei_in_stroma = get_objs(stroma_roi, nuc, predicate="contains")

### **Spatial Partitioning**

from histolytics.spatial_ops.ops import get_interfaces

from histolytics.spatial_ops.h3 import h3_grid

from histolytics.spatial_ops.rect_grid import rect_grid

from histolytics.data import cervix_tissue # Example data

tissue = cervix_tissue()

stroma = tissue[tissue[“class_name”] == “stroma”]]

lesion = tissue[tissue[“class_name”] == “cin”]]

#Partition stroma with hexagonal and rectangular grid
hex_grid = h3_grid(stroma, resolution=8)
rectangular_grid = rect_grid(stroma, resolution=(512,512))

# Partition stroma into lesion-stroma interface

LSI = get_interface(lesion, stroma, buffer_dist=200)

# References

[1] Pourakpour F, Szölgyén Á, Nateghi R, Gutman DA, Manthey D, Cooper LA. HistomicsTK: A Python toolkit for pathology image analysis algorithms. SoftwareX 2025;31:102318. <https://doi.org/10.1016/j.softx.2025.102318>.

[2] Pocock J, Graham S, Vu QD, Jahanifar M, Deshpande S, Hadjigeorghiou G, et al. TIAToolbox as an end-to-end library for advanced tissue image analytics. Communications Medicine 2022;2:120. <https://doi.org/10.1038/s43856-022-00186-5>.

[3] Dolezal JM, Kochanny S, Dyer E, Ramesh S, Srisuwananukorn A, Sacco M, et al. Slideflow: deep learning for digital histopathology with real-time whole-slide visualization. BMC Bioinformatics 2024;25:134. <https://doi.org/10.1186/s12859-024-05758-x>.

[4] Rosenthal J, Carelli R, Omar M, Brundage D, Halbert E, Nyman J, et al. Building Tools for Machine Learning and Artificial Intelligence in Cancer Research: Best Practices and a Case Study with the PathML Toolkit for Computational Pathology. Molecular Cancer Research 2022;20:202–6. <https://doi.org/10.1158/1541-7786.MCR-21-0665>.

[5] Palla G, Spitzer H, Klein M, Fischer D, Schaar AC, Kuemmerle LB, et al. Squidpy: a scalable framework for spatial omics analysis. Nat Methods 2022;19:171–8. <https://doi.org/10.1038/s41592-021-01358-2>.

[6] Nirmal AJ, Sorger PK. SCIMAP: A Python Toolkit for Integrated Spatial Analysis of Multiplexed Imaging Data. J Open Source Softw 2024;9:6604. <https://doi.org/10.21105/joss.06604>.

[7] Amgad M, Hodge JM, Elsebaie MAT, Bodelon C, Puvanesarajah S, Gutman DA, et al. A population-level digital histologic biomarker for enhanced prognosis of invasive breast cancer. Nat Med 2024;30:85–97. <https://doi.org/10.1038/s41591-023-02643-7>.

[8] Faust K, Chen ML, Babaei Zadeh P, Oreopoulos DG, Leon AJ, Paliwal A, et al. PHARAOH: A collaborative crowdsourcing platform for phenotyping and regional analysis of histology. Nat Commun 2025;16:742. <https://doi.org/10.1038/s41467-024-55780-z>.

[9] Gamper J, Alemi Koohbanani N, Benet K, Khuram A, Rajpoot N. PanNuke: An Open Pan-Cancer Histology Dataset for Nuclei Instance Segmentation and Classification, 2019, p. 11–9. <https://doi.org/10.1007/978-3-030-23937-4_2>.

[10] Gamper J, Koohbanani NA, Benes K, Graham S, Jahanifar M, Khurram SA, et al. PanNuke Dataset Extension, Insights and Baselines 2020.

[11] Bankhead P, Loughrey MB, Fernández JA, Dombrowski Y, McArt DG, Dunne PD, et al. QuPath: Open source software for digital pathology image analysis. Sci Rep 2017;7:16878. <https://doi.org/10.1038/s41598-017-17204-5>.
